# Association of prognostic nutritional index with the risk of all-cause mortality and cardiovascular events in patients with diabetes-related foot ulcers: a non-linear relationship mediated by eGFR

**DOI:** 10.3389/fnut.2026.1825009

**Published:** 2026-06-10

**Authors:** Yukun Tao, Lulu Liu, Jinzheng Hou, Li Xiao, Huijuan Zhu, Guangxin Zhou, Maolin Tian, Di Zhu, Da Zhang

**Affiliations:** Department of Endocrinology, Air Force Medical Center, Air Force Medical University, Beijing, China

**Keywords:** all-cause mortality, cardiovascular mortality, diabetes-related foot ulcer (DFU), eGFR, major adverse cardiovascular events (MACE), prognostic nutritional index (PNI)

## Abstract

**Background:**

Diabetes-related foot ulcers (DFU), a severe complication of diabetes, is strongly associated with high mortality. Although the prognostic nutritional index (PNI) has been validated for predicting clinical outcomes, its precise relationship with mortality risk in patients with DFU remains unclear. This study aimed to investigate the association between PNI and all-cause mortality, cardiovascular mortality, and major adverse cardiovascular events (MACE) in patients with DFU, and to elucidate the underlying non-linear mechanisms.

**Methods:**

This retrospective cohort study consecutively enrolled 1,225 patients with DFU admitted to Air Force Medical Center from January 2015 to December 2022. Cox proportional hazards models were used to calculate HR with 95% CI for evaluating the association of PNI with all endpoints and Kaplan–Meier survival curves were generated. Restricted cubic spline (RCS) evaluated non-linear associations, and piecewise regression identified risk thresholds. Causal mediation analysis quantified the mediating effect of eGFR.

**Results:**

During a median follow-up of 3.6 years, 378 (30.9%) all-cause deaths, 230 (18.8%) cardiovascular deaths, and 323 (26.4%) MACEs occurred. Multivariable-adjusted Cox proportional hazards models revealed significant inverse associations between PNI and all endpoints. In the fully adjusted model, each 1-unit increase in PNI was associated with a 5% reduction in all-cause mortality risk (HR: 0.95, 95%CI: 0.94–0.97, *p* < 0.001), a 4% reduction in cardiovascular mortality risk (HR: 0.96, 95%CI: 0.94–0.98, *p* < 0.001), and a 3% reduction in MACE risk (HR: 0.97, 95%CI: 0.95–0.98, *p* < 0.001). Quartile analysis revealed progressively decreasing risks across Q2 to Q4 groups versus Q1 (lowest quartile) for all endpoints. Kaplan–Meier survival curves corroborated the Cox regression results. RCS analysis demonstrated significant non-linear relationships between PNI and all endpoints, with exploratory, cohort-specific thresholds identified near 39.29–39.37 (non-linear *p* < 0.05). Causal mediation analysis revealed that eGFR mediated 11.5 and 11.9% of the effects of PNI on the risks of all-cause mortality and cardiovascular mortality, respectively (*p* < 0.05).

**Conclusion:**

Prognostic nutritional index is an independent protective factor associated with lower risks of all-cause mortality, cardiovascular mortality, and MACE in patients with DFU. Elevated PNI is associated with significantly reduced risks of all-cause mortality and cardiovascular events, with eGFR partially mediating this association.

## 1 Introduction

Diabetes mellitus, a globally prevalent chronic metabolic disorder, poses a substantial public health burden due to its high rates of mortality and disability (1, 2). Driven by large-scale urbanization and population aging, the number of individuals with diabetes is escalating dramatically. Current estimates indicate a global prevalence of 537 million, projected to rise to 783 million by 2045 (3). Among the complications of diabetes mellitus, diabetes-related foot ulcers (DFU) stands out as one of the most common and severe (4). DFU is associated not only with high incidence but also with an elevated mortality risk and substantial socioeconomic costs (5). A large prospective cohort study revealed that patients with DFU have a median survival of only 7.72 years, with mortality rates reaching 22% at 5 years and 71% at 10 years, significantly higher than in diabetic patients without DFU (5-year mortality: 3%) (6). Other studies corroborate these findings, reporting 5-year mortality rates of up to 30.5% in patients with DFU, a severity comparable to certain cancers (7). Notably, cardiovascular events represent the primary complication during DFU management, occurring frequently—particularly within the first 10 days post-admission or post-surgery—and are characterized by sudden onset and high mortality (8). Therefore, the precise identification and effective intervention of key factors influencing all-cause mortality and cardiovascular event risk in patients with DFU are crucial for improving survival rates.

Emerging evidence underscores the significant role of nutritional status in the development and progression of DFU. Malnutrition is prevalent among DFU patients (occurring in approximately 62%), a rate significantly higher than in diabetic patients without DFU (9). Identifying this high-risk patient subgroup with potential malnutrition is critical, as they may derive substantial benefit from clinical nutritional interventions, leading to improved prognosis and extended survival (10).

Prognostic nutritional index (PNI), a novel composite nutritional and inflammatory marker calculated from serum albumin levels and lymphocyte counts, reflects chronic inflammation, immune function, and nutritional status (11). Originally proposed by Onodera et al., PNI has gained widespread use for prognostic assessment in various diseases due to its ease of calculation (12). A low PNI has been robustly associated with adverse outcomes in diverse conditions, including cardiovascular diseases and malignancies (13). Particularly relevant is the evidence that low PNI levels significantly increase the risk of all-cause mortality and cardiovascular disease-related mortality in patients with type 2 diabetes mellitus and gestational diabetes mellitus (14, 15). However, the prognostic value of PNI in the high-risk DFU population remains incompletely understood. The precise association between PNI and mortality in patients with DFU, along with its predictive power for risk stratification, warrants further investigation.

To address this gap in the literature, this study employed diverse analytical approaches to comprehensively investigate the potential associations between PNI and the risks of all-cause mortality, cardiovascular mortality, and major adverse cardiovascular events (MACE) in patients with DFU.

## 2 Materials and methods

### 2.1 Study population

**Figure 1:**
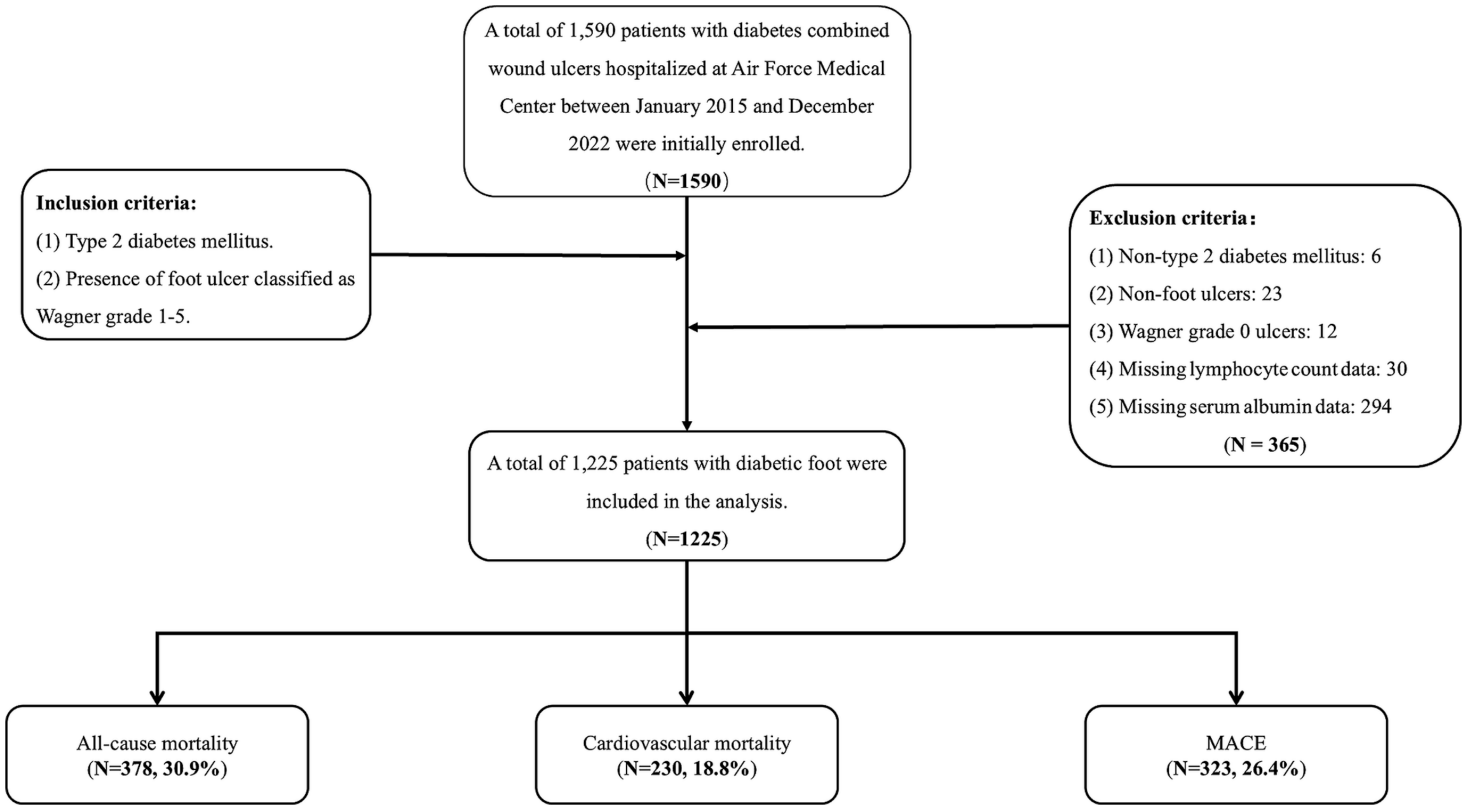
Flow chart of patient selection.

This was a single-center retrospective cohort study. Data were extracted from the medical records of 1,590 patients with diabetes combined wound ulcers hospitalized at Air Force Medical Center between January 2015 and December 2022. Inclusion Criteria: (1) Type 2 diabetes mellitus (16); (2) Presence of foot ulcer classified as Wagner grade 1–5. Exclusion Criteria: (1) Non-type 2 diabetes mellitus (*n* = 6); (2) Non-foot ulcers (*n* = 23); (3) Wagner grade 0 ulcers (*n* = 12); (4) Missing lymphocyte count data (*n* = 30); (5) Missing serum albumin data (*n* = 294). A total of 1,225 patients with DFU were ultimately included in the final analysis. The study was conducted in accordance with the principles of the Declaration of Helsinki and approved by the Institutional Review Board of Air Force Medical Center (Approval No: AFMC-2024-64-PJ01). A waiver of informed consent was granted. Reporting strictly followed the Strengthening the Reporting of Observational Studies in Epidemiology (STROBE) guidelines. The patient selection flow chart is presented in Figure 1.

### 2.2 Follow-up and outcomes

Clinical follow-up was conducted from December 2023 to March 2024, yielding a median follow-up duration of 3.6 years. Patient survival status was ascertained by trained, experienced clinicians using standardized procedures via telephone interviews, text messages, and systematic review of the hospital’s electronic medical records system. Mortality data, including vital status and specific causes of death, were further verified by linkage with the Chinese Center for Disease Control and Prevention database.

The study endpoints were defined as follows: All-cause mortality was defined as death from any cause occurring from the date of the first hospitalization for DFU until the end of follow-up. Cardiovascular mortality referred to death directly attributable to cardiovascular disease, defined according to relevant International Classification of Diseases, Tenth Revision (ICD-10) codes: I00-I09, I11, I13, I20-I51, and I60-I69 (17). MACE constituted a composite endpoint (18) defined as the occurrence of any of the following: (a) Cardiovascular mortality (as defined above); (b) Non-fatal myocardial infarction, referring to acute myocardial necrosis but not resulting in death, accompanied by ischemia symptoms, abnormal myocardial markers, ST-segment changes or pathological Q waves; (c) Non-fatal stroke, defined as a focal neurological deficit of cerebrovascular origin lasting >24 h, confirmed by brain imaging (computed tomography or magnetic resonance imaging) as acute cerebral infarction, intracerebral hemorrhage, or subarachnoid hemorrhage without resulting in death.

### 2.3 Assessment of PNI

The PNI was used to assess nutritional status, and calculated using the formula (19): PNI = 5 × lymphocyte count (10^9^/L) + serum albumin (g/L). Serum albumin concentration was measured using the bromocresol green method on Beckman Coulter AU5800 automated analyzer. Lymphocyte count was analyzed in peripheral blood samples using the fluorescence flow cytometry method with nucleic acid staining on Sysmex XN-9100 hematology analyzer. Based on the calculated PNI values, participants were stratified into quartiles (Q): Q1 (PNI ≤ 40.5), Q2 (40.5 < PNI ≤ 45.7), Q3 (45.7 < PNI ≤ 49.7), and Q4 (PNI > 49.7).

### 2.4 Assessment of covariates

The same set of covariates was considered for analyses of all-cause mortality, cardiovascular mortality, and MACE. Baseline data were collected from Air Force Medical Center electronic medical records system and included: (1) Demographics: age (at the time of first hospitalization for DFU diagnosis), sex, BMI (calculated as weight in kilograms divided by height in meters squared), smoking status (dichotomized as current smoker vs. never/former smoker). (2) Disease course, complications and comorbidities: diabetes duration (years, from initial type 2 diabetes mellitus diagnosis to death/last follow-up), DFU duration (months, from initial DFU diagnosis to death/last follow-up), diabetic peripheral neuropathy (DPN), diabetic retinopathy (DR), diabetic nephropathy (DN), peripheral artery disease (PAD), hypertension, CHD, stroke, hyperlipidemia, Wagner grade (grade 1: superficial ulcers without clinical infection; grade 2: deep ulcer without bone tissue lesion or abscess; grade 3: deep ulcer with bone tissue lesion or abscess; grade 4: localized gangrene (toe, heel, or forefoot); grade 5: whole foot gangrene). All complications and comorbidities were dichotomized as present or absent. (3) Laboratory Parameters (measured at first DF hospitalization): white blood cell count (WBC), hemoglobin (HB), platelet (PLT), lymphocyte count, monocyte count, HbA1c, fasting blood glucose (FBG), blood urea nitrogen (BUN), serum creatinine (SCr), serum uric acid (SUA), triglycerides, HDL cholesterol, LDL cholesterol, total protein, serum albumin, C-reactive protein (CRP), eGFR [calculated using the Chronic Kidney Disease Epidemiology Collaboration (CKD-EPI) equation (20)].

### 2.5 Statistical analysis

In this study, patients were stratified into quartiles (Q1–Q4) based on PNI values. Descriptive statistics were used to summarize baseline characteristics across PNI quartiles. The normality of continuous variables was assessed using the Kolmogorov–Smirnov test. Normally distributed variables were expressed as mean ± standard deviation and compared using the *t*-test or the ANOVA test. Non-normally distributed variables were presented as median (IQR) and compared using the Mann–Whitney *U* test or the Kruskal–Wallis *H* test. Categorical variables were analyzed by calculating frequencies and percentages [*n*(%)] and compared using the Chi-square test or the Fisher exact test.

Missing data, a common issue in clinical research that may compromise data integrity and robustness, were observed across some variables. Under the assumption of missing at random, multiple imputation was performed using the random forest algorithm. All subsequent analyses utilized the imputed dataset. To assess the robustness of the results, we performed a sensitivity analysis using complete-case analysis (i.e., restricting the analysis to participants with complete data on all covariates).

Univariable and multivariable Cox proportional hazards regression models were constructed to evaluate associations between PNI and risks of all-cause mortality, cardiovascular mortality, and MACE. Model 1: Crude model (unadjusted); Model 2: Adjusted for age and sex; Model 3: Fully adjusted for age, sex, BMI, smoking status, diabetes duration, DFU duration, DPN, DR, DN, PAD, hypertension, CHD, stroke, hyperlipidemia, and Wagner grade. Results are presented as HR with 95% CI. Proportional hazards assumptions were assessed globally and for each covariate using Schoenfeld residual tests in the initial unstratified Cox models. The test revealed that DPN violated the proportional hazards assumption (*p* < 0.05). Therefore, DPN was treated as a stratification variable rather than a covariate in all final models. After stratification by DPN, no remaining variable violated the proportional hazards assumption in any of the three outcome-specific models (all global *p* > 0.05; see Supplementary Table S1). Kaplan–Meier (KM) curves visualized survival differences across PNI quartiles, with log-rank tests assessing statistical significance.

Restricted cubic splines (RCS) with four knots were fitted using the R rms package (v8.0–0) to examine potential non-linear dose–response relationships between PNI and risks of all-cause mortality, cardiovascular mortality, and MACE. Non-linearity was tested via the ANOVA likelihood ratio tests. Threshold effects were explored using piecewise regression models. It is important to note that the HRs derived from the linear Cox models represent average effects across the range of PNI.

To investigate potential heterogeneity in the associations of PNI with risks of all-cause mortality, cardiovascular mortality and MACE across clinically relevant subgroups, subgroup analyses were conducted according to the following eight stratification variables: age (≤65 years or >65 years), sex (male or female), BMI (≤24 kg/m^2^, 24–28 kg/m^2^ or >28 kg/m^2^), diabetes duration (≤10 years or >10 years), PAD (yes or no), CHD (yes or no), smoking (yes or no), eGFR (CKD1, CKD2, CKD3 or CKD4-5). Statistical interaction was assessed using likelihood ratio tests, with the *p*-value for interaction reported. Given potential limitations related to sample size and multiple comparisons, findings from subgroup analyses should be interpreted as exploratory.

Causal mediation analysis quantified the mediating effect of eGFR in the association between PNI and all-cause mortality, cardiovascular mortality and MACE, estimating the average causal mediation effect, average direct effect, and proportion mediated. This analysis is exploratory and hypothesis-generating, and does not establish causality due to the observational study design. All analyses used two-sided tests with statistical significance set at *p* < 0.05 and were performed in R (v4.4.2; www.r-project.org).

## 3 Results

### 3.1 Baseline characteristics

This study enrolled 1,225 patients with DFU. Table 1 presents the baseline demographic and clinical characteristics of the patients stratified by PNI quartile groups. Overall, the cohort comprised 886 (72.3%) males. The median age was 63 years (IQR: 55, 70), median BMI was 24.22 kg/m^2^ (IQR: 22.20, 26.78), and median PNI was 45.7 (IQR: 40.5, 49.7). Patients in the lowest PNI quartile (Q1 patients) exhibited significantly worse clinical and metabolic profiles compared to those in the highest quartile (Q4 patients). Specifically, Q1 patients had a significantly higher prevalence of DN (70% vs. 46%, *p* < 0.001), higher proportion of advanced Wagner grades (grade 4–5: 73% vs. 36%, *p* < 0.001), and poorer glycemic control (FBG: 8.3 mmol/L vs. 7.2 mmol/L, *p* < 0.001; HbA1c: 9.4% vs. 8.3%, *p* < 0.001). Furthermore, Q1 patients demonstrated poorer nutritional status, with significantly lower serum albumin, total protein, and HB (*p* < 0.001). They also exhibited higher inflammatory markers, evidenced by significantly elevated CRP and WBC (*p* < 0.001). Renal function parameters were worse in Q1 patients, with higher SCr and lower eGFR (*p* < 0.05). Statistically significant differences (*p* < 0.05) were also observed across PNI quartiles for PLT, SUA, triglycerides, HDL cholesterol, LDL cholesterol, and BMI. Supplementary Table S2 presents baseline characteristics stratified by the outcomes of all-cause mortality, cardiovascular mortality, and MACE. Over a median follow-up of 3.6 years, 378 (30.9%) all-cause deaths, 230 (18.8%) cardiovascular deaths, and 323 (26.4%) MACEs were recorded. Patients experiencing any of these adverse outcomes were generally older and had significantly higher prevalence of hypertension, CHD, stroke, and DN, as well as significantly higher BUN, SCr, and CRP compared to those without the respective events. Conversely, eGFR, serum albumin, and PNI were significantly lower in patients experiencing these outcomes. Variance inflation factor (VIF) analysis indicated no substantial multicollinearity among the variables included in the analyses (all VIF < 10; see Supplementary Table S3).

**Table 1:** Baseline clinical characteristics of patients according to quartiles of PNI.

Indicators	Total	Q1 ≤ 40.5	40.5 < Q2 ≤ 45.7	45.7 < Q3 ≤ 49.7	Q4 > 49.7	*p*
*N*	1,225	307	306	308	304	
PNI	45.70 (40.50, 49.70)	37.40 (34.77, 39.10)	43.40 (42.20, 44.70)	47.55 (46.63, 48.50)	52.25 (50.80, 54.70)	<0.001
Age (years)	63 (55, 70)	62 (55, 70)	64 (55, 71)	64 (56, 69)	62 (54, 69)	0.135
Sex, *n* (%)						0.22
Male	886 (72)	235 (77)	221 (72)	213 (69)	217 (71)	
Female	339 (28)	72 (23)	85 (28)	95 (31)	87 (29)	
BMI (kg/m^2^)	24.22 (22.20, 26.78)	24.21 (21.96, 26.63)	23.94 (22.02, 26.63)	24.06 (22.20, 26.85)	24.98 (22.76, 27.52)	0.031
Smoking, *n* (%)						0.179
No	711 (58)	177 (58)	192 (63)	178 (58)	164 (54)	
Yes	514 (42)	130 (42)	114 (37)	130 (42)	140 (46)	
Diabetes duration (years)	15 (8, 20)	15 (9, 20)	15 (10, 20)	15 (8, 20)	15 (8, 20)	0.974
DFU duration (months)	30 (20, 100)	30 (20, 90)	30 (20, 90)	35.5 (20, 100)	60 (20.75, 120)	0.011
DPN, *n* (%)						0.657
No	147 (12)	42 (14)	32 (10)	38 (12)	35 (12)	
Yes	1,078 (88)	265 (86)	274 (90)	270 (88)	269 (88)	
DR, *n* (%)						0.104
No	592 (48)	164 (53)	134 (44)	152 (49)	142 (47)	
Yes	633 (52)	143 (47)	172 (56)	156 (51)	162 (53)	
DN, *n* (%)						<0.001
No	403 (44)	62 (30)	98 (42)	116 (49)	127 (54)	
Yes	508 (56)	143 (70)	136 (58)	119 (51)	110 (46)	
PAD, *n* (%)						0.912
No	351 (29)	86 (28)	84 (27)	91 (30)	90 (30)	
Yes	874 (71)	221 (72)	222 (73)	217 (70)	214 (70)	
Hypertension, *n* (%)						0.172
No	379 (31)	84 (27)	95 (31)	92 (30)	108 (36)	
Yes	846 (69)	223 (73)	211 (69)	216 (70)	196 (64)	
CHD, *n* (%)						0.934
No	888 (72)	223 (73)	221 (72)	227 (74)	217 (71)	
Yes	337 (28)	84 (27)	85 (28)	81 (26)	87 (29)	
Stroke, *n* (%)						0.41
No	972 (79)	253 (82)	242 (79)	237 (77)	240 (79)	
Yes	253 (21)	54 (18)	64 (21)	71 (23)	64 (21)	
Hyperlipidemia, *n* (%)						<0.001
No	362 (30)	121 (39)	89 (29)	81 (26)	71 (23)	
Yes	863 (70)	186 (61)	217 (71)	227 (74)	233 (77)	
Wagner grade, *n* (%)						<0.001
1	51 (4)	3 (1)	7 (2)	16 (5)	25 (8)	
2	180 (15)	20 (7)	37 (12)	48 (16)	75 (25)	
3	335 (27)	59 (19)	78 (25)	103 (33)	95 (31)	
4–5	659 (54)	225 (73)	184 (60)	141 (46)	109 (36)	
WBC (10^9/L)	7.40 (5.86, 9.50)	9 (6.40, 12.54)	7.40 (5.70, 9.2)	6.70 (5.50, 8.68)	7 (6, 8.40)	<0.001
HB (g/L)	113.50 ± 20.19	98.82 ± 16.91	110.25 ± 17.64	117.94 ± 18.06	127.10 ± 16.74	<0.001
PLT (10^9/L)	272 (207.75, 351)	314 (239, 404)	278 (211.25, 357.75)	262 (195, 328)	241.50 (200, 305.25)	<0.001
Lymphocyte count (10^9/L)	1.60 (1.30, 2)	1.22 (1, 1.50)	1.52 (1.20, 1.80)	1.70 (1.40, 1.97)	2.10 (1.70, 2.50)	<0.001
Monocyte count (10^9/L)	0.50 (0.40, 0.70)	0.58 (0.40, 0.8)	0.50 (0.40, 0.70)	0.49 (0.40, 0.60)	0.50 (0.40, 0.60)	<0.001
HbA1c (%)	8.70 (7.40, 10.3)	9.40 (7.75, 10.95)	9.10 (7.60, 10.50)	8.40 (7, 9.80)	8.30 (7.30, 9.60)	<0.001
FBG (mmol/L)	7.90 (6.07, 10.60)	8.30 (6.08, 11.90)	8.50 (6.30, 10.92)	7.70 (6.10, 9.80)	7.20 (5.80, 9.70)	<0.001
BUN (mmol/L)	5.90 (4.50, 7.90)	5.50 (4, 8.35)	5.90 (4.60, 7.90)	5.80 (4.50, 8.03)	6.20 (4.90, 7.45)	0.135
SCr (umol/L)	70 (56, 92.78)	71.40 (56, 101.45)	72 (58, 96.90)	70 (55.50, 91.50)	67 (56, 81)	0.036
SUA (umol/L)	296 (233, 369)	270 (205, 343)	283.50 (228.25, 359)	308 (248, 373.25)	318.50 (259, 385.75)	<0.001
Triglycerides (mmol/L)	1.26 (0.97, 1.72)	1.12 (0.90, 1.49)	1.20 (0.91, 1.64)	1.31 (1.04, 1.75)	1.48 (1.1, 2.12)	<0.001
HDL cholesterol (mmol/L)	0.91 (0.74, 1.10)	0.77 (0.62, 0.97)	0.92 (0.77, 1.11)	0.93 (0.79, 1.13)	0.98 (0.83, 1.14)	<0.001
LDL cholesterol (mmol/L)	2.19 (1.66, 2.76)	1.98 (1.52, 2.56)	2.29 (1.70, 2.83)	2.21 (1.73, 2.74)	2.29 (1.70, 2.88)	<0.001
Total protein (g/L)	66.41 ± 6.60	61.47 ± 6.39	65.91 ± 5.72	67.80 ± 5.53	70.47 ± 5.26	<0.001
Albumin (g/L)	37.40 (33.30, 40.40)	30.90 (28.05, 32.70)	35.80 (34.20, 37.48)	39.10 (37.70, 40.42)	42.35 (40.40, 44.40)	<0.001
CRP (mg/L)	11.60 (3.35, 50)	59 (20.47, 105.81)	15.11 (4.88, 58)	7.05 (2.75, 20.66)	3.98 (2, 9)	<0.001
eGFR (ml/min/1.73m^2^)	92.60 (71.30, 105.09)	92.25 (63.57, 106.58)	89.93 (67.90, 101.87)	92.38 (72.78, 104.34)	95.35 (79.04, 107.62)	0.004

PNI, prognostic nutritional index; DFU, diabetes-related foot ulcers; DPN, diabetic peripheral neuropathy; DR, diabetic retinopathy; DN, diabetic nephropathy; PAD, peripheral artery disease; WBC, white blood cell count; HB, hemoglobin; PLT, platelet; FBG, fasting blood glucose; BUN, blood urea nitrogen; SCr, serum creatinine; SUA, serum uric acid; CRP, C-reactive protein.

Data are presented as mean ± SD, *N* (%) or median (IQR).

Using the original data for baseline description, missing values of covariates in 1225 patients were as follows: BMI 228(18.6%), DN 314(25.6%), WBC 3(0.2%), PLT 1(0.08%), monocyte count 1(0.08%), HbA1c 233(19.0%), FBG 3(0.2%), BUN 2(0.2%), SCr 5(0.4%), SUA 2(0.2%), triglycerides 3(0.2%), HDL cholesterol 3(0.2%), LDL cholesterol 4(0.3%), total protein 4(0.3%), CRP 72(5.9%), eGFR 5(0.4%).

### 3.2 The association between PNI and all-cause mortality, cardiovascular mortality and MACE

**Figure 2:**
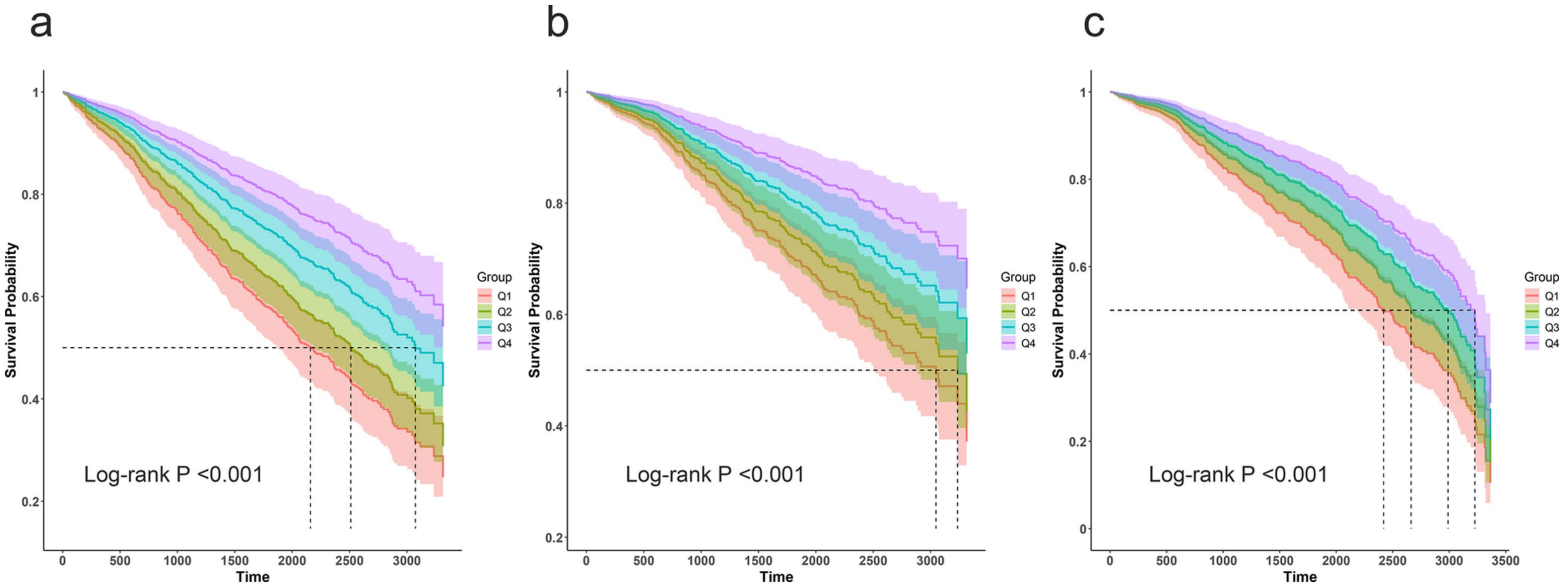
Kaplan–Meier survival curves for all-cause mortality **(a)**, cardiovascular mortality **(b)**, and MACE **(c)** by PNI quartiles.

Univariable and multivariable Cox proportional hazards regression analyses demonstrated a consistent and statistically significant inverse association between the PNI and the risks of all-cause mortality, cardiovascular mortality, and MACE in patients with DFU (Table 2). In the fully adjusted model (Model 3), the hazard ratio represents the average effect of PNI across its entire range. However, given the non-linear relationship identified by RCS analysis (see below), the actual magnitude of association between PNI and each outcome varies substantially depending on the baseline PNI level. Specifically, each 1-unit increase in continuous PNI was associated with a 5% reduction in the risk of all-cause mortality (HR:0.95, 95% CI:0.94–0.97, *p* < 0.001), a 4% reduction in the risk of cardiovascular mortality (HR: 0.96, 95% CI: 0.94–0.98, *p* < 0.001), and a 3% reduction in the risk of MACE (HR: 0.97, 95% CI: 0.95–0.98, *p* < 0.001). When analyzed by PNI quartile groups, compared to Q1 patients, Q4 patients exhibited a 63% lower risk of all-cause mortality (HR: 0.37, 95% CI: 0.26–0.51, *p* for trend < 0.001), a 61% lower risk of cardiovascular mortality (HR: 0.39, 95% CI: 0.25–0.60, *p* for trend < 0.001), and a 53% lower risk of MACE (HR: 0.47, 95% CI: 0.33–0.67, *p* for trend < 0.001). KM survival curves based on Model 3 (Figure 2) revealed significant differences in survival probability for all-cause mortality, cardiovascular mortality, and MACE across the PNI quartile groups (log-rank test: *p* < 0.001 for all). Compared to patients in the lower PNI quartiles (Q1–Q3 patients), Q4 patients experienced significantly prolonged survival time and higher survival probability.

**Table 2:** Cox proportional hazard models for the association of PNI and all-cause mortality, cardiovascular mortality and MACE.

Indicators	Model 1		Model 2		Model 3	
	HR (95%CI)	*p*-value	HR (95%CI)	*P*-value	HR (95%CI)	*P*-value
All-cause mortality						
PNI (Continuous)	0.95 (0.94, 0.97)	<0.001	0.96 (0.94, 0.97)	<0.001	0.95 (0.94, 0.97)	<0.001
PNI (Quartiles)						
Q1	Reference		Reference		Reference	
Q2	0.82 (0.63, 1.06)	0.130	0.78 (0.60, 1.00)	0.058	0.80 (0.62, 1.04)	0.101
Q3	0.55 (0.42, 0.74)	<0.001	0.57 (0.43, 0.76)	<0.001	0.54 (0.40, 0.73)	<0.001
Q4	0.38 (0.28, 0.52)	<0.001	0.41 (0.30, 0.56)	<0.001	0.37 (0.26, 0.51)	<0.001
*P* for trend		<0.001		<0.001		<0.001
Cardiovascular mortality						
PNI (Continuous)	0.95 (0.94, 0.97)	<0.001	0.96 (0.94, 0.97)	<0.001	0.96 (0.94, 0.98)	<0.001
PNI (Quartiles)						
Q1	Reference		Reference		Reference	
Q2	0.81 (0.58, 1.14)	0.226	0.78 (0.56, 1.09)	0.147	0.84 (0.59, 1.18)	0.307
Q3	0.60 (0.42, 0.86)	0.005	0.60 (0.42, 0.86)	0.006	0.59 (0.40, 0.87)	0.007
Q4	0.39 (0.26, 0.58)	<0.001	0.40 (0.27, 0.60)	<0.001	0.39 (0.25, 0.60)	<0.001
*P* for trend		<0.001		<0.001		<0.001
MACE						
PNI (Continuous)	0.97 (0.95, 0.98)	<0.001	0.97 (0.95, 0.98)	<0.001	0.97 (0.95, 0.98)	<0.001
PNI (Quartiles)						
Q1	Reference		Reference		Reference	
Q2	0.80 (0.59, 1.07)	0.133	0.77 (0.57, 1.03)	0.083	0.80 (0.59, 1.08)	0.137
Q3	0.66 (0.49, 0.90)	0.008	0.66 (0.49, 0.90)	0.009	0.64 (0.46, 0.88)	0.007
Q4	0.48 (0.34, 0.66)	<0.001	0.49 (0.35, 0.68)	<0.001	0.47 (0.33, 0.67)	<0.001
*P* for trend		<0.001		<0.001		<0.001

Model 1: crude model (unadjusted).

Model 2: adjust for: age; sex.

Model 3: adjust for: age, sex, BMI, smoking, diabetes duration, DFU duration, DPN, DR, DN, PAD, hypertension, CHD, stroke, hyperlipidemia, Wagner grade.

**Figure 3:**
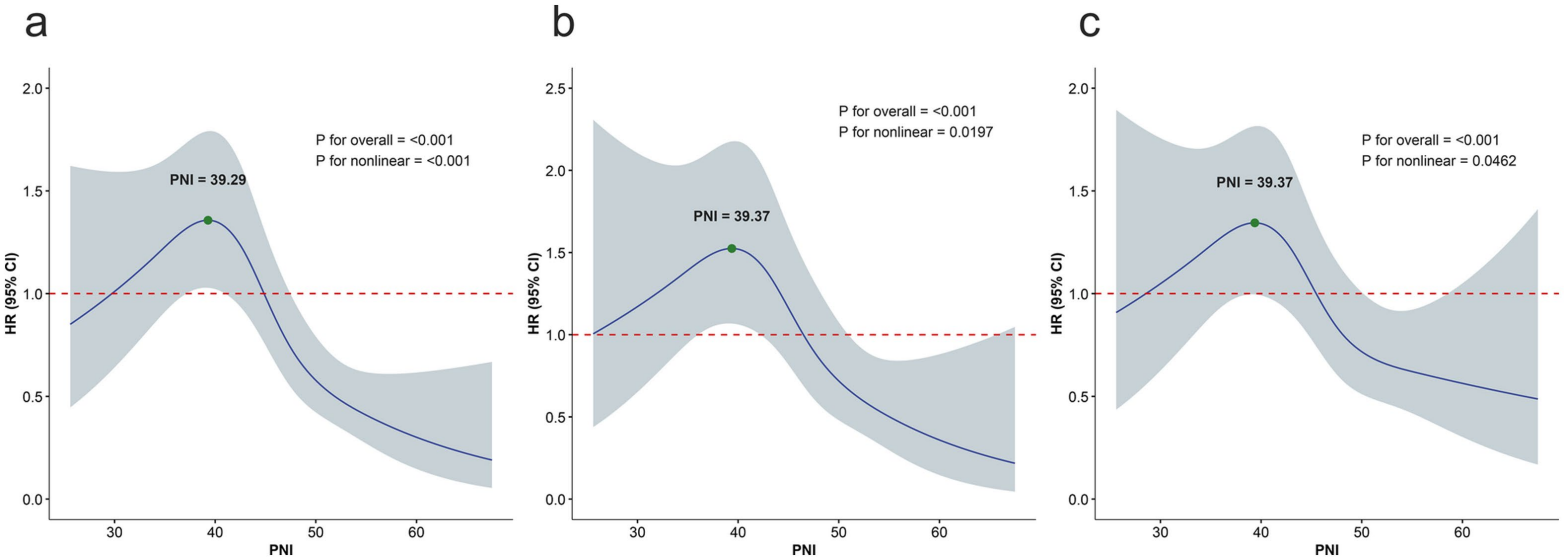
Restricted cubic spline curves of the association between PNI and all-cause mortality **(a)**, cardiovascular mortality **(b)**, and MACE **(c)**. According to age, sex, BMI, smoking, diabetes duration, DFU duration, DPN, DN, DR, PAD, hypertension, CHD, stroke, hyperlipidemia, Wagner grade to adjust.

The RCS analysis (Figure 3) further elucidated a non-linear dose–response relationship between PNI and the risks of all-cause mortality, cardiovascular mortality, and MACE. Under the fully adjusted multivariable model (Model 3), significant non-linear associations were observed between PNI and the risks of all-cause mortality (non-linearity *p* < 0.001), cardiovascular mortality (non-linearity *p* = 0.0197), and MACE (non-linearity *p* = 0.0462). The identified inflection points on the curves, 39.3 for all-cause mortality, and 39.4 for cardiovascular mortality and MACE, suggest the presence of potential exploratory, cohort-specific threshold effects. Piecewise regression analysis (see Supplementary Table S4) demonstrated that when PNI exceeded this exploratory threshold, each 1-unit increase in PNI was associated with an 8% reduction in the risk of all-cause mortality (HR: 0.92, 95% CI: 0.89–0.94, *p* < 0.001), an 8% reduction in the risk of cardiovascular mortality (HR: 0.92, 95% CI: 0.90–0.96, *p* < 0.001), and a 6% reduction in the risk of MACE (HR: 0.94, 95% CI: 0.92–0.97, *p* < 0.001).

### 3.3 Subgroup analysis

**Figure 4:**
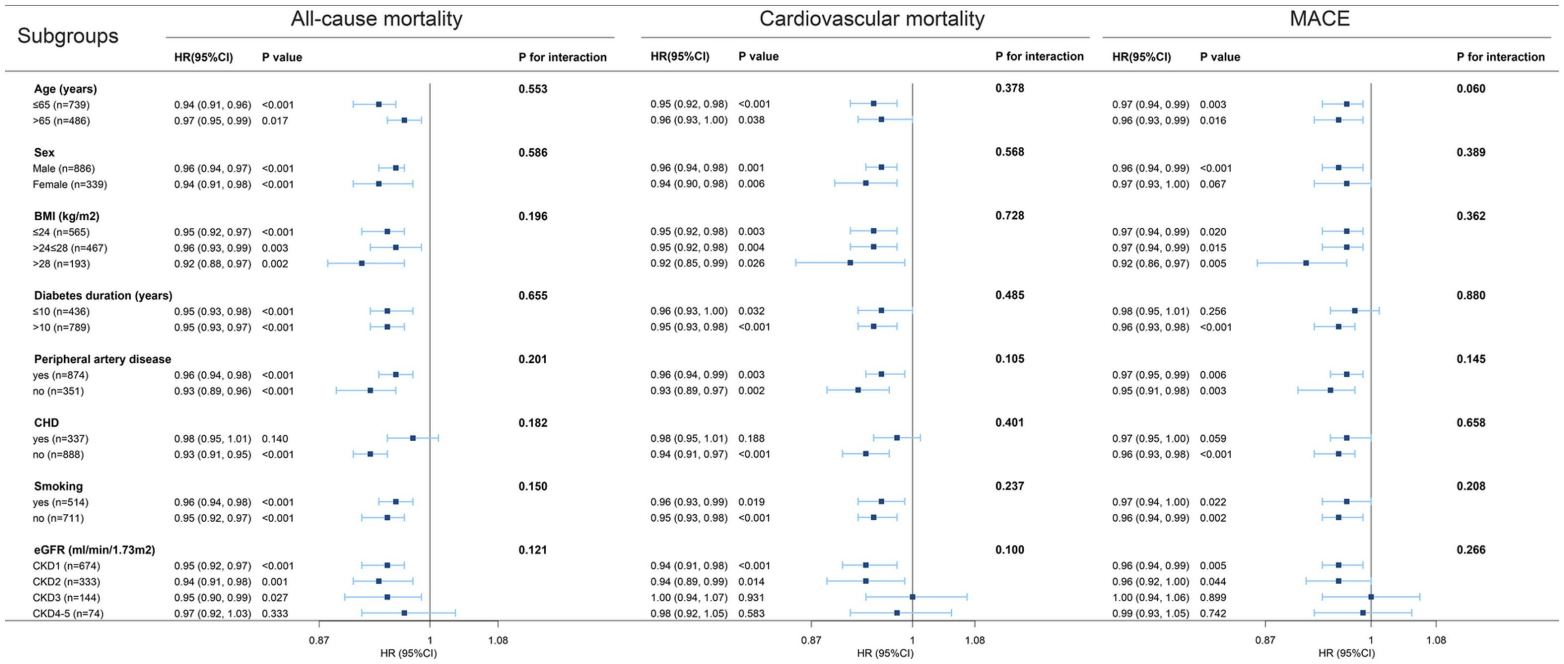
Subgroup analysis for the all-cause mortality, cardiovascular mortality, and MACE. The stratifications were adjusted for all variables (age, sex, BMI, smoking, diabetes duration, DFU duration, DPN, DR, DN, PAD, hypertension, CHD, stroke, hyperlipidemia, Wagner grade) except for the stratification factor itself.

Results of the subgroup analyses (Figure 4) consistently demonstrated a significant inverse association between the PNI and the risks of all-cause mortality, cardiovascular mortality, and MACE across all subgroups (all *p* < 0.05). No significant interaction effects were observed between PNI and any subgroup variable for the risks of these three outcomes (all *P* for interaction > 0.05) (Figure 4).

### 3.4 Causal mediation analysis

**Figure 5:**
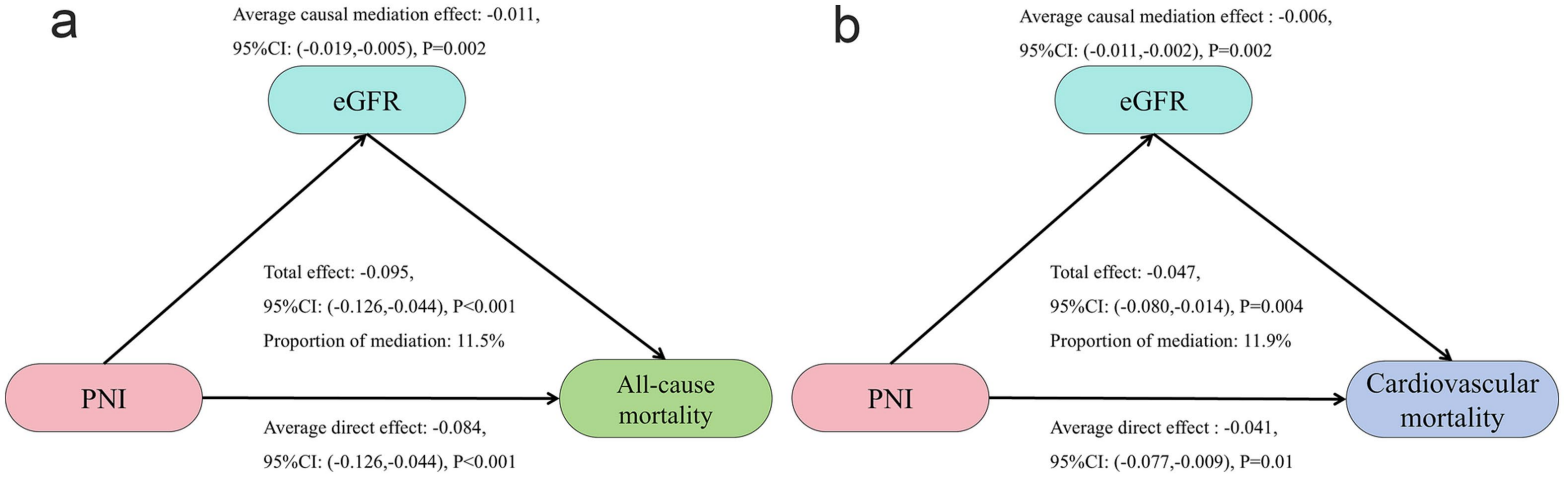
Causal mediation analysis for all-cause mortality **(a)** and cardiovascular mortality **(b)**.

Causal mediation analysis revealed that eGFR significantly mediated the associations between PNI and both all-cause and cardiovascular mortality (Figure 5). In this exploratory analysis, eGFR accounted for 11.5% (95% CI: 5.1–23.7%; *p* = 0.002) of the total effect of PNI on all-cause mortality. For cardiovascular mortality, the mediation proportion via eGFR was 11.9% (95% CI: 3.0–41.9%; *p* = 0.006). However, no significant mediating effect of eGFR was identified in the association between PNI and MACE (*p* > 0.05).

### 3.5 Sensitivity analysis

To assess the robustness of our findings, we repeated the fully adjusted Cox models using complete-case analysis (*n* = 609). The results were highly consistent with the primary analysis, and all conclusions remained unchanged (see Supplementary Table S5).

## 4 Discussion

This study is the first to investigate the association between the PNI and the risks of all-cause mortality, cardiovascular mortality, and MACE in patients with DFU. We demonstrated significant inverse associations between PNI and all three outcomes. Quartile analysis revealed a clear dose–response relationship. Compared to patients in the lowest quartile (Q1), those in Q2–Q4 exhibited progressively greater risk reductions (*P* for trend <0.05), with the maximal reduction observed in Q4 patients. Subgroup analyses further confirmed the robustness and validity of these findings. RCS analysis identified significant non-linear relationships (*p* < 0.05) and critical thresholds. Risk decreased significantly above these thresholds. Importantly, this study revealed that eGFR partially mediated the associations of PNI on all-cause mortality and cardiovascular mortality, establishing a novel renal-mediated pathway: PNI → eGFR → Outcomes.

Consistent with expectations, PNI showed expected correlations with its individual components (serum albumin and lymphocyte count). As shown in Table 1, patients in the lowest PNI quartile also had significantly higher levels of inflammatory markers (CRP, WBC), reflecting the interplay between nutritional status and systemic inflammation.

Numerous studies have established significant associations between PNI and prognosis in various conditions, including coronary heart disease (21), type 2 diabetes mellitus and its complications (22, 23), malignancies (24, 25) and post-fracture outcomes (26). Particularly in patients with diabetes, low PNI is consistently recognized as a predictor of increased all-cause mortality and cardiovascular events. Ning et al. (14) demonstrated that low serum PNI was associated with significantly elevated all-cause mortality and cardiovascular mortality in a study of 5,916 patients with diabetes. Xu et al. (27) also found inverse, non-linear (L-shaped) relationships between PNI and risks of all-cause and cardiovascular mortality in patients with diabetes or prediabetes. Our findings align with the literature.

In a study of 386 patients with DFU (stratified into amputation and non-amputation groups), Coşkun et al. (28) identified PNI as a strong predictor of amputation (AUC = 0.937), with a critical threshold of <39.005 conferring an 81.8-fold increased amputation risk. This threshold closely corresponds to the risk thresholds for all-cause and cardiovascular mortality identified in our study. Bing Sun et al. revealed an association between PNI and sepsis in DF, confirming PNI as an independent predictor; PNI ≤ 34.75 was associated with significantly increased sepsis risk (29). Hong et al. (30) comparatively evaluated three nutritional indices—Geriatric Nutritional Risk Index, PNI, and Controlling Nutritional Status—for predicting all-cause mortality in patients with DFU. They confirmed PNI as an independent predictor, with PNI < 43.6 associated with a 2.04-fold increased mortality risk (HR = 2.04). Our study not only corroborates these conclusions but further elucidates the associations between PNI and cardiovascular mortality and MACE risk, providing a crucial foundation for future large-scale prospective studies aimed at improving DFU patient outcomes.

The RCS analysis revealed a notable non-monotonic pattern at very low PNI values (below approximately 39), where the lowest PNI values were not associated with the highest estimated risk, but rather with a comparatively moderate hazard ratio. This pattern is likely attributable to sparse data in the extreme low PNI range, model instability, or residual confounding. From a clinical perspective, the curve suggests that risk reduction accelerates above a PNI of approximately 39, indicating a potential threshold effect. Clinically, this implies that efforts to raise PNI above this level may yield greater prognostic benefit than small increments at lower levels. However, this pattern requires confirmation in larger cohorts with more patients in the very low PNI range.

Although the precise biological mechanisms linking PNI to renal function decline are not fully elucidated, several theoretical frameworks exist. PNI is significantly influenced by nutritional status and systemic inflammation. Inflammation or malnutrition can reduce serum albumin levels, and hypoalbuminemia is strongly associated with worsening renal function (31, 32), potentially mediated by promoting endothelial damage and glomerulosclerosis (33). Lymphocytes, conversely, play key roles in inflammatory regulation and protection (34). Evidence suggests that pharmacologically induced increases in lymphocyte count are associated with slowed chronic kidney disease (CKD) progression (35), while lymphopenia exacerbates renal microinflammation, fibrosis, and accelerates diabetic kidney disease (36). Malnutrition and inflammation independently induce endothelial dysfunction, oxidative stress, and impaired immune responses—pathological processes implicated in CKD pathogenesis (37, 38). Furthermore, inadequate nutrient intake, particularly protein-energy malnutrition, may lead to muscle wasting and reduced renal perfusion, potentially exacerbating renal injury over time (39). A large cohort study (*n* = 15,437) by In Park et al. (40) investigating PNI and CKD found that the low PNI group exhibited significantly greater eGFR decline over 5 years compared to the highest PNI group. Reduced eGFR not only reflects renal dysfunction but also signifies underlying pathologies like inflammation, microangiopathy, and enhanced oxidative stress—common pathways contributing to cardiovascular disease, CKD progression, and ultimately increased all-cause mortality (41). Additionally, eGFR decline can promote vascular medial calcification, valvular calcification, and left ventricular hypertrophy via effects on circulatory volume and pressure homeostasis, thereby increasing cardiovascular mortality risk (42).

In this exploratory analysis, eGFR partially mediated the associations between PNI and both all-cause and cardiovascular mortality, with mediation proportions of 11.5 and 11.9%, respectively. This modest proportion suggests that eGFR is only one of several potential pathways through which PNI may influence these outcomes. The proposed renal-mediated pathway (PNI → eGFR → Outcomes) should therefore be considered hypothesis-generating rather than definitive causal evidence. Due to the observational study design, causality cannot be inferred from this mediation analysis. Future prospective studies with repeated measurements and interventional designs are needed to validate these findings.

Notably, we did not observe a significant mediating effect of eGFR on the association between PNI and MACE (*p* > 0.05). The composite MACE endpoint includes outcomes with distinct pathophysiological mechanisms (cardiovascular mortality, non-fatal myocardial infarction, non-fatal stroke). The absence of significant mediation by eGFR for the composite MACE may reflect aggregation bias rather than a true lack of mediation, as eGFR could exert differential effects across individual MACE components, which cannot be captured in a composite analysis. Future studies should analyze individual MACE components separately to disentangle these pathways.

The results of this observational study suggest a potential clinical utility of PNI in DFU patient management, offering a simple, objective biomarker for risk stratification. However, the identified PNI threshold of 39.4 is exploratory and cohort-specific, derived from a single baseline PNI measurement within a single-center cohort. Given that PNI is a dynamic parameter influenced by inflammatory and nutritional status, a single measurement may not adequately reflect patients’ longitudinal risk profiles. Therefore, this threshold should not be directly generalized to other populations (e.g., civilians and community hospitals) without independent external validation. Furthermore, based on the identified “PNI → eGFR → Outcomes” pathway, which remains exploratory, we propose that future prospective studies investigate the potential value of dynamic combined monitoring of PNI and eGFR. These findings provide hypothesis-generating evidence for developing innovative clinical pathways to improve the prognosis of patients with DFU.

Our study cohort was drawn from a single tertiary hospital, with a predominance of male patients (72.3%), which differs substantially from the general DFU population. This demographic characteristic combined with the single-center design may limit generalizability to populations with different sex distributions or healthcare settings. Furthermore, our cohort had a high proportion of advanced Wagner grades (grades 4–5: 54%), which may limit generalizability to patients with milder DFU. Therefore, the findings, particularly the specific PNI threshold of 39.4, should be generalized with caution to civilian, community-based, or more sex-balanced DFU populations. External validation in diverse, multi-center cohorts is essential before clinical application.

This study has several notable strengths. First, the use of diverse analytical methods, standardized outcomes adjudication, and comprehensive adjustment for covariates enhance the robustness of the findings. Second, this is the first study to reveal the potential mediating role of eGFR in the associations between PNI and all-cause mortality risk and cardiovascular mortality risk in patients with DFU.

Despite these strengths, several limitations warrant consideration. First, the retrospective cohort design cannot fully eliminate residual confounding or potential biases that may influence causal inference. Second, unmeasured confounding factors may persist. In particular, we lacked data on limb ischemia (e.g., ankle-brachial index, skin perfusion pressure), which is an important prognostic factor in DFU and could influence both nutritional status and outcomes. Third, PNI was measured only once at baseline and reflects a single time point. PNI is a dynamic parameter influenced by inflammation, infection, and nutritional interventions. The lack of serial measurements precludes assessment of temporal changes and their potential impact on outcomes. Forth, the identified PNI threshold of approximately 39.4 is exploratory and derived from a single-center cohort; it requires independent external validation before generalization. Future research should address these limitations by expanding sample sizes, incorporating more comprehensive clinical and biomarker data, and conducting well-designed prospective studies to validate these findings and further elucidate the underlying biological mechanisms.

## 5 Conclusion

This study demonstrates that the PNI is associated with a significant independent protective factor against all-cause mortality, cardiovascular mortality, and MACE in patients with DFU. Higher PNI levels were significantly associated with reduced risks of all-cause mortality and cardiovascular events, exhibiting a clear dose–response relationship. Furthermore, a non-linear threshold effect was identified; above the exploratory, cohort-specific threshold, each 1-unit increase in PNI was associated with a significantly greater magnitude of reduction in risks of all-cause mortality and cardiovascular events. Additionally, the study revealed that eGFR partially mediated the associations of PNI on all-cause and cardiovascular mortality. Due to the observational study design, causality cannot be inferred. Interventional studies are needed to determine whether improving PNI causally improves outcomes. These findings provide hypothesis-generating evidence for interventions targeting improved nutritional status and renal function to enhance clinical outcomes in patients with DFU.

## Data Availability

The raw data supporting the conclusions of this article will be made available by the authors, without undue reservation.
