# Supplementary figures and images for: Association of prognostic nutritional index with the risk of all-cause mortality and cardiovascular events in patients with diabetes-related foot ulcers: a non-linear relationship mediated by eGFR
Source: Front Nutr. 2026 Jun 10;13:1825009. doi: 10.3389/fnut.2026.1825009 (PMC13290545; doi:10.3389/fnut.2026.1825009)

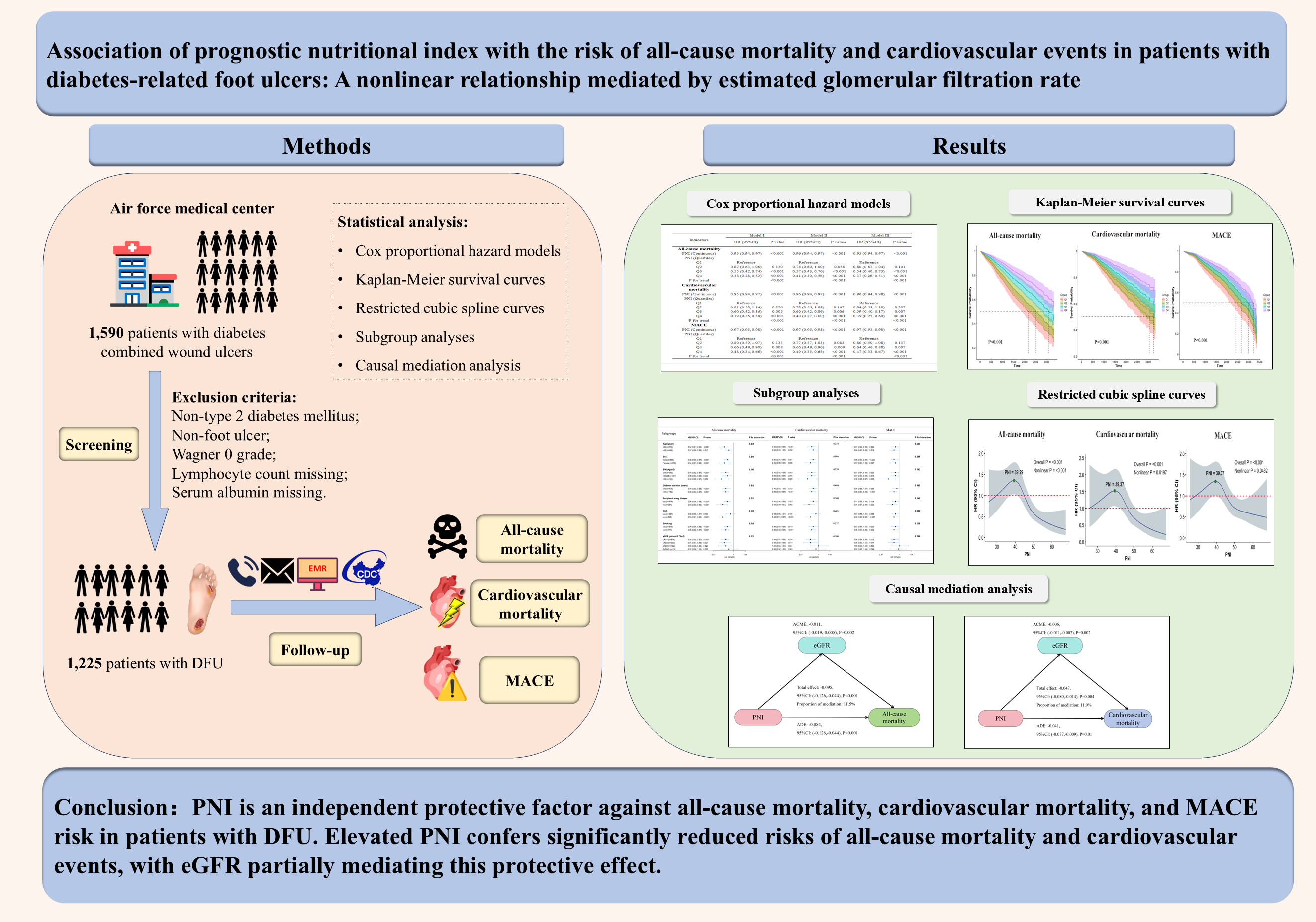

Supplement: Supplementary file 1 [file Image_1.TIF]
