# Supplementary material for: Association of prognostic nutritional index with the risk of all-cause mortality and cardiovascular events in patients with diabetes-related foot ulcers: a non-linear relationship mediated by eGFR
Source: Front Nutr. 2026 Jun 10;13:1825009. doi: 10.3389/fnut.2026.1825009 (PMC13290545; doi:10.3389/fnut.2026.1825009)
Supplement: Supplementary file 2 [file Table_1.DOCX]

**Table S1** Schoenfeld residual test results for proportional hazards assumption in the three final stratified Cox models.

| Variable | All-cause mortality  chisq (df) / p | Cardiovascular mortality  chisq (df) / p | MACE  chisq (df) / p |
| --- | --- | --- | --- |
| Age | 0.07 (1) / 0.79 | 0.07 (1) / 0.79 | 0.96 (1) / 0.33 |
| Sex | 0.71 (1) / 0.40 | 0.15 (1) / 0.70 | 0.44 (1) / 0.51 |
| BMI | 0.51 (1) / 0.47 | 1.22 (1) / 0.27 | 2.70 (1) / 0.10 |
| Diabetes duration | 1.58 (1) / 0.21 | <0.01 (1) / 0.99 | <0.01 (1) / 0.98 |
| DFU duration | 0.08 (1) / 0.78 | 0.08 (1) / 0.78 | 0.76 (1) / 0.38 |
| PAD | 0.36 (1) / 0.55 | 0.18 (1) / 0.67 | 0.20 (1) / 0.65 |
| DR | 2.06 (1) / 0.15 | 0.01 (1) / 0.91 | 0.09 (1) / 0.76 |
| Hypertension | 0.03 (1) / 0.87 | 0.16 (1) / 0.69 | 0.48 (1) / 0.49 |
| CHD | 1.67 (1) / 0.20 | 0.11 (1) / 0.74 | 0.02 (1) / 0.90 |
| Stroke | 0.03 (1) / 0.86 | 0.64 (1) / 0.42 | 0.93 (1) / 0.34 |
| Hyperlipidemia | 0.18 (1) / 0.67 | <0.01 (1) / 0.99 | 1.19 (1) / 0.27 |
| Smoking | 0.12 (1) / 0.73 | 0.09 (1) / 0.77 | 0.02 (1) / 0.90 |
| DN | 0.15 (1) / 0.69 | 0.47 (1) / 0.49 | 1.99 (1) / 0.16 |
| Wagner grade | 1.80 (3) / 0.62 | 2.54 (3) / 0.47 | 6.00 (3) / 0.11 |
| GLOBAL | 16.40 (19) / 0.63 | 7.98 (19) / 0.99 | 23.50 (19) / 0.22 |

*DPN was used as a stratification variable in all models and therefore is not included in the proportional hazards test. Stratification corrects for violation of the proportional hazards assumption by DPN without requiring estimation of its coefficient.*

**Table S2** Baseline clinical characteristics of patients stratified by all-cause mortality, cardiovascular mortality and MACE.

| Variables | Total | All-cause mortality | | | Cardiovascular mortality | | | MACE | | |
| --- | --- | --- | --- | --- | --- | --- | --- | --- | --- | --- |
|  |  | Survival | Death | p | Survival | Death | p | Non-MACE | MACE | p |
| N | 1225 | 847 | 378 |  | 995 | 230 |  | 902 | 323 |  |
| Age (years) | 63 (55, 70) | 61 (52, 68) | 66 (60, 73) | < 0.001 | 62 (54, 69) | 65 (60, 72) | < 0.001 | 62 (54, 70) | 64 (58, 70) | 0.003 |
| Sex, n (%) |  |  |  | 0.771 |  |  | 0.641 |  |  | 0.987 |
| Male | 886 (72) | 610 (72) | 276 (73) |  | 723 (73) | 163 (71) |  | 653 (72) | 233 (72) |  |
| Female | 339 (28) | 237 (28) | 102 (27) |  | 272 (27) | 67 (29) |  | 249 (28) | 90 (28) |  |
| BMI (kg/m^2^) | 24.22 (22.2, 26.78) | 24.22 (22.3, 26.77) | 24.42 (22.04, 26.89) | 0.571 | 24.22 (22.16, 26.77) | 24.62 (22.4, 27.06) | 0.77 | 24.19 (22.15, 26.69) | 24.66 (22.45, 27.24) | 0.141 |
| Smoking, n (%) |  |  |  | 0.388 |  |  | 0.373 |  |  | 0.291 |
| no | 711 (58) | 499 (59) | 212 (56) |  | 571 (57) | 140 (61) |  | 515 (57) | 196 (61) |  |
| yes | 514 (42) | 348 (41) | 166 (44) |  | 424 (43) | 90 (39) |  | 387 (43) | 127 (39) |  |
| Diabetes duration (years) | 15 (8, 20) | 15 (8, 20) | 15 (10, 20) | 0.15 | 15 (8, 20) | 15 (10, 20) | 0.128 | 15 (8, 20) | 15 (10, 20) | 0.055 |
| DFU duration (months) | 30 (20, 100) | 30 (20, 95) | 30 (20, 115) | 0.722 | 30 (20, 100) | 30 (20, 90) | 0.376 | 30 (20, 100) | 30 (20, 100) | 0.94 |
| DPN, n (%) |  |  |  | 1 |  |  | 0.839 |  |  | 0.395 |
| no | 147 (12) | 102 (12) | 45 (12) |  | 118 (12) | 29 (13) |  | 113 (13) | 34 (11) |  |
| yes | 1078 (88) | 745 (88) | 333 (88) |  | 877 (88) | 201 (87) |  | 789 (87) | 289 (89) |  |
| DR, n (%) |  |  |  | 0.471 |  |  | 0.959 |  |  | 0.468 |
| no | 592 (48) | 403 (48) | 189 (50) |  | 480 (48) | 112 (49) |  | 442 (49) | 150 (46) |  |
| yes | 633 (52) | 444 (52) | 189 (50) |  | 515 (52) | 118 (51) |  | 460 (51) | 173 (54) |  |
| DN, n (%) |  |  |  | 0.008 |  |  | 0.006 |  |  | 0.001 |
| no | 403 (44) | 304 (47) | 99 (37) |  | 348 (46) | 55 (34) |  | 321 (47) | 82 (35) |  |
| yes | 508 (56) | 341 (53) | 167 (63) |  | 402 (54) | 106 (66) |  | 356 (53) | 152 (65) |  |
| PAD, n (%) |  |  |  | 0.015 |  |  | 0.3 |  |  | 0.662 |
| no | 351 (29) | 261 (31) | 90 (24) |  | 292 (29) | 59 (26) |  | 262 (29) | 89 (28) |  |
| yes | 874 (71) | 586 (69) | 288 (76) |  | 703 (71) | 171 (74) |  | 640 (71) | 234 (72) |  |
| Hypertension, n (%) |  |  |  | 0.006 |  |  | 0.005 |  |  | 0.001 |
| no | 379 (31) | 283 (33) | 96 (25) |  | 326 (33) | 53 (23) |  | 303 (34) | 76 (24) |  |
| yes | 846 (69) | 564 (67) | 282 (75) |  | 669 (67) | 177 (77) |  | 599 (66) | 247 (76) |  |
| CHD, n (%) |  |  |  | < 0.001 |  |  | < 0.001 |  |  | < 0.001 |
| no | 888 (72) | 652 (77) | 236 (62) |  | 748 (75) | 140 (61) |  | 685 (76) | 203 (63) |  |
| yes | 337 (28) | 195 (23) | 142 (38) |  | 247 (25) | 90 (39) |  | 217 (24) | 120 (37) |  |
| Stroke, n (%) |  |  |  | < 0.001 |  |  | 0.001 |  |  | 0.004 |
| no | 972 (79) | 701 (83) | 271 (72) |  | 808 (81) | 164 (71) |  | 734 (81) | 238 (74) |  |
| yes | 253 (21) | 146 (17) | 107 (28) |  | 187 (19) | 66 (29) |  | 168 (19) | 85 (26) |  |
| Hyperlipidemia, n (%) |  |  |  | 0.664 |  |  | 0.231 |  |  | 0.323 |
| no | 362 (30) | 254 (30) | 108 (29) |  | 302 (30) | 60 (26) |  | 274 (30) | 88 (27) |  |
| yes | 863 (70) | 593 (70) | 270 (71) |  | 693 (70) | 170 (74) |  | 628 (70) | 235 (73) |  |
| Wagner grade, n (%) |  |  |  | 0.695 |  |  | 0.772 |  |  | 0.784 |
| 1 | 51 (4) | 34 (4) | 17 (4) |  | 40 (4) | 11 (5) |  | 37 (4) | 14 (4) |  |
| 2 | 180 (15) | 120 (14) | 60 (16) |  | 149 (15) | 31 (13) |  | 136 (15) | 44 (14) |  |
| 3 | 335 (27) | 239 (28) | 96 (25) |  | 276 (28) | 59 (26) |  | 251 (28) | 84 (26) |  |
| 4-5 | 659 (54) | 454 (54) | 205 (54) |  | 530 (53) | 129 (56) |  | 478 (53) | 181 (56) |  |
| WBC (10^9/L) | 7.4 (5.86, 9.5) | 7.2 (5.8, 9.3) | 7.58 (5.9, 9.7) | 0.221 | 7.3 (5.8, 9.5) | 7.8 (5.9, 9.65) | 0.119 | 7.2 (5.8, 9.51) | 7.8 (6, 9.38) | 0.033 |
| HB (g/L) | 113.5 ± 20.19 | 115.38 ± 19.77 | 109.3 ± 20.53 | < 0.001 | 114.35 ± 20.09 | 109.85 ± 20.26 | 0.002 | 113.94 ± 20.18 | 112.28 ± 20.23 | 0.207 |
| PLT (10^9/L) | 272 (207.75, 351) | 274.5 (210, 352) | 264.5 (198.25, 344) | 0.137 | 271 (205.5, 350.75) | 274 (215, 351) | 0.647 | 271.5 (207, 349) | 273.5 (212, 353.5) | 0.981 |
| Lymphocyte count (10^9/L) | 1.6 (1.3, 2) | 1.63 (1.3, 2.08) | 1.5 (1.2, 1.88) | < 0.001 | 1.6 (1.3, 2) | 1.6 (1.2, 1.95) | 0.179 | 1.6 (1.3, 2) | 1.6 (1.2, 2) | 0.84 |
| Monocyte count (10^9/L) | 0.5 (0.4, 0.7) | 0.5 (0.4, 0.68) | 0.5 (0.4, 0.7) | 0.149 | 0.5 (0.4, 0.69) | 0.5 (0.4, 0.7) | 0.351 | 0.5 (0.4, 0.69) | 0.5 (0.4, 0.7) | 0.233 |
| HbA1c (%) | 8.7 (7.4, 10.3) | 8.7 (7.4, 10.2) | 8.6 (7.2, 10.5) | 0.893 | 8.7 (7.4, 10.3) | 8.7 (7.32, 10.5) | 0.482 | 8.7 (7.4, 10.3) | 8.7 (7.4, 10.4) | 0.495 |
| FBG (mmol/L) | 7.9 (6.07, 10.6) | 8 (6.1, 10.5) | 7.8 (6, 11) | 0.86 | 7.8 (6, 10.5) | 8.1 (6.4, 11.4) | 0.062 | 7.7 (5.9, 10.3) | 8.5 (6.4, 11.34) | 0.004 |
| BUN (mmol/L) | 5.9 (4.5, 7.9) | 5.6 (4.4, 7.3) | 6.5 (5.1, 9.3) | < 0.001 | 5.8 (4.5, 7.68) | 6.3 (4.9, 8.8) | 0.003 | 5.7 (4.5, 7.7) | 6.15 (4.8, 8.3) | 0.016 |
| SCr (umol/L) | 70 (56, 92.78) | 68 (55, 85) | 77 (59, 114) | < 0.001 | 69 (56, 89) | 76.5 (57.75, 112) | < 0.001 | 69 (56, 88) | 75 (57, 107) | < 0.001 |
| SUA (umol/L) | 296 (233, 369) | 293 (230, 366.5) | 299 (235, 374) | 0.289 | 296 (232.25, 368) | 294 (234, 374) | 0.799 | 296 (231, 370) | 295 (240, 364.75) | 0.632 |
| Triglycerides (mmol/L) | 1.26 (0.97, 1.72) | 1.26 (0.99, 1.76) | 1.23 (0.94, 1.7) | 0.242 | 1.25 (0.97, 1.69) | 1.33 (0.99, 1.81) | 0.237 | 1.24 (0.97, 1.67) | 1.33 (0.99, 1.87) | 0.048 |
| HDL cholesterol (mmol/L) | 0.91 (0.74, 1.1) | 0.9 (0.74, 1.07) | 0.92 (0.75, 1.16) | 0.028 | 0.91 (0.75, 1.09) | 0.9 (0.74, 1.15) | 0.465 | 0.91 (0.75, 1.09) | 0.89 (0.74, 1.13) | 0.729 |
| LDL cholesterol (mmol/L) | 2.19 (1.66, 2.76) | 2.2 (1.65, 2.74) | 2.17 (1.66, 2.84) | 0.619 | 2.2 (1.65, 2.74) | 2.19 (1.7, 2.91) | 0.104 | 2.18 (1.65, 2.73) | 2.22 (1.67, 2.9) | 0.127 |
| Total protein (g/L) | 66.41 ± 6.6 | 66.76 ± 6.4 | 65.61 ± 6.99 | 0.005 | 66.48 ± 6.38 | 66.1 ± 7.51 | 0.432 | 66.38 ± 6.38 | 66.5 ± 7.2 | 0.773 |
| Albumin (g/L) | 37.4 (33.3, 40.4) | 37.9 (33.8, 40.9) | 36.4 (32.52, 39.4) | < 0.001 | 37.6 (33.6, 40.8) | 36.25 (32.12, 39.3) | < 0.001 | 37.65 (33.5, 40.7) | 36.9 (32.75, 39.65) | 0.01 |
| CRP (mg/L) | 11.6 (3.35, 50) | 11.2 (3.01, 46.99) | 12.2 (4.7, 57.57) | 0.043 | 10.72 (3, 48.95) | 14 (5.77, 55.4) | 0.011 | 10.4 (3, 49.7) | 13.75 (4.99, 52.67) | 0.018 |
| eGFR (ml/min/1.73m^2^) | 92.6 (71.3, 105.09) | 95.12 (78.75, 107.67) | 83.44 (54.56, 98.11) | < 0.001 | 93.43 (75.13, 106.21) | 86.85 (57.45, 99.47) | < 0.001 | 93.5 (75.64, 106.58) | 88.78 (60, 101.42) | < 0.001 |
| PNI | 45.7 (40.5, 49.7) | 46.5 (41.11, 50.38) | 44.2 (39.5, 47.64) | < 0.001 | 46.1 (40.83, 50) | 44.27 (39.56, 47.8) | < 0.001 | 46.08 (40.7, 49.98) | 45 (40.4, 48.4) | 0.021 |

DFU: diabetes-related foot ulcers; DPN: diabetic peripheral neuropathy; DR: diabetic retinopathy; DN: diabetic nephropathy; PAD: peripheral artery disease; WBC: white blood cell count; HB: hemoglobin; PLT: platelet; FBG: fasting blood glucose; BUN: blood urea nitrogen; SCr: serum creatinine; SUA: serum uric acid; CRP: C-reactive protein; PNI: prognostic nutritional index.

Data are presented as mean ± SD, N (%) or median (IQR).

**Table S3** Collinearity diagnostics among the variables.

| Variables | VIF |
| --- | --- |
| Age | 2.271201 |
| Sex | 1.667883 |
| BMI | 1.27475 |
| Smoking | 1.395805 |
| Diabetes duration | 1.304315 |
| DFU duration | 1.101735 |
| DPN | 1.087191 |
| DR | 1.115296 |
| DN | 1.207641 |
| PAD | 1.179765 |
| Hypertension | 1.398753 |
| CHD | 1.249965 |
| Stroke | 1.143311 |
| Hyperlipidemia | 1.26764 |
| Wagner grade | 1.476875 |
| WBC | 3.390736 |
| HB | 2.0319 |
| PLT | 1.641018 |
| Lymphocyte count | 1.41352 |
| Monocyte count | 2.217391 |
| HbA1c | 1.469117 |
| FBG | 1.277942 |
| BUN | 2.98961 |
| SCr | 3.1057 |
| SUA | 1.669785 |
| Triglycerides | 1.431906 |
| HDL cholesterol | 1.478382 |
| LDL cholesterol | 1.238568 |
| Total protein | 1.658278 |
| Albumin | 2.662381 |
| CRP | 2.40714 |
| eGFR | 4.128132 |

VIF: variance inflation factor. Other abbreviations are listed in Table S1.

No potentially significant collinearity is defined as VIF less than 10 among variables.

**Table S4** Segment regression for all-cause mortality, cardiovascular mortality and MACE.

| Indicators | HR (95%CI) | P value |
| --- | --- | --- |
| **All-cause mortality** |  |  |
| Straight-line effect | 0.96 (0.94, 0.98) | < 0.001 |
| Segment 1：<39.3 | 1.06 (1.00, 1.11) | 0.054 |
| Segment 2：>39.3 | 0.92 (0.89, 0.94) | < 0.001 |
| Likelihood ratio test | < 0.001 | |
|  |  | |
| **Cardiovascular mortality** |  |  |
| Straight-line effect | 0.96 (0.94, 0.98) | < 0.001 |
| Segment 1：<39.4 | 1.05 (0.98, 1.12) | 0.189 |
| Segment 2：>39.4 | 0.92 (0.90, 0.96) | < 0.001 |
| Likelihood ratio test | 0.003 | |
|  |  |  |
| **MACE** |  |  |
| Straight-line effect | 0.96 (0.94, 0.98) | < 0.001 |
| Segment 1：<39.4 | 1.04 (0.98, 1.10) | 0.241 |
| Segment 2：>39.4 | 0.94 (0.92, 0.97) | < 0.001 |
| Likelihood ratio test | < 0.001 | |

**Table S5** Sensitivity analysis: Association of PNI with all-cause mortality, cardiovascular mortality, and MACE using complete-case analysis (excluding participants with missing covariates).

| Indicators | Model 1 | | Model 2 | | Model 3 | |
| --- | --- | --- | --- | --- | --- | --- |
|  | HR (95%CI) | P value | HR (95%CI) | P valuse | HR (95%CI) | P value |
| **All-cause mortality** |  |  |  |  |  |  |
| PNI (Continuous) | 0.95 (0.92, 0.97) | <0.001 | 0.96 (0.93, 0.98) | <0.001 | 0.94 (0.92, 0.97) | <0.001 |
| PNI (Quartiles) |  |  |  |  |  |  |
| Q1 | Reference |  | Reference |  | Reference |  |
| Q2 | 0.76 (0.52, 1.11) | 0.158 | 0.78 (0.53, 1.14) | 0.201 | 0.73 (0.49, 1.08) | 0.116 |
| Q3 | 0.46 (0.30, 0.71) | <0.001 | 0.54 (0.35, 0.84) | 0.007 | 0.49 (0.30, 0.78) | 0.003 |
| Q4 | 0.32 (0.20, 0.53) | <0.001 | 0.42 (0.25, 0.69) | <0.001 | 0.33 (0.19, 0.57) | <0.001 |
| P for trend |  | <0.001 |  | <0.001 |  | <0.001 |
| **Cardiovascular mortality** |  |  |  |  |  |  |
| PNI (Continuous) | 0.94 (0.91, 0.97) | <0.001 | 0.95 (0.91, 0.98) | <0.001 | 0.94 (0.91, 0.97) | <0.001 |
| PNI (Quartiles) |  |  |  |  |  |  |
| Q1 | Reference |  | Reference |  | Reference |  |
| Q2 | 0.80 (0.49, 1.32) | 0.380 | 0.82 (0.50, 1.35) | 0.425 | 0.82 (0.49, 1.38) | 0.464 |
| Q3 | 0.53 (0.30, 0.92) | 0.024 | 0.58 (0.33, 1.03) | 0.061 | 0.55 (0.30, 1.01) | 0.054 |
| Q4 | 0.28 (0.14, 0.55) | <0.001 | 0.34 (0.17, 0.68) | 0.002 | 0.30 (0.14, 0.63) | 0.001 |
| P for trend |  | <0.001 |  | <0.001 |  | <0.001 |
| **MACE** |  |  |  |  |  |  |
| PNI (Continuous) | 0.96 (0.93, 0.98) | 0.001 | 0.96 (0.94, 0.99) | 0.005 | 0.96 (0.93, 0.99) | 0.003 |
| PNI (Quartiles) |  |  |  |  |  |  |
| Q1 | Reference |  | Reference |  | Reference |  |
| Q2 | 0.87 (0.56, 1.34) | 0.524 | 0.88 (0.57, 1.36) | 0.575 | 0.92 (0.59, 1.44) | 0.719 |
| Q3 | 0.59 (0.37, 0.94) | 0.027 | 0.64 (0.40, 1.03) | 0.065 | 0.59 (0.36, 0.98) | 0.041 |
| Q4 | 0.48 (0.29, 0.79) | 0.004 | 0.55 (0.33, 0.92) | 0.023 | 0.51 (0.30, 0.89) | 0.018 |
| P for trend |  | <0.001 |  | <0.001 |  | <0.001 |

*Complete-case analysis included 609 participants with no missing data on any covariate.

Model 1: crude model (unadjusted).

Model 2: adjust for: age; sex.
Model 3: adjust for: age, sex, BMI, smoking, diabetes duration, DFU duration, DPN, DR, DN, PAD, hypertension, CHD, stroke, hyperlipidemia, Wagner grade.
